# Risk factors for the development of opioid use disorder after first opioid prescription: a Swedish national study

**DOI:** 10.1017/S003329172200349X

**Published:** 2023-10

**Authors:** Kenneth S. Kendler, Sara L. Lönn, John Ektor-Andersen, Jan Sundquist, Kristina Sundquist

**Affiliations:** 1 Virginia Institute for Psychiatric and Behavioral Genetics, Virginia Commonwealth University, Richmond, VA, USA; 2 Department of Psychiatry, Virginia Commonwealth University, Richmond, VA, USA; 3 Center for Primary Health Care Research, Lund University, Malmö, Sweden; 4 Department of Family Medicine and Community Health, Department of Population Health Science and Policy, Icahn School of Medicine at Mount Sinai, New York, USA; 5 Department of Clinical Sciences Lund, Psychiatry, Faculty of Medicine, Lund University, Lund, Sweden and Addiction Center Malmö, Division of Psychiatry, Malmö, Sweden

**Keywords:** Follow-up study, opioid prescription, opioid use disorder, Sweden

## Abstract

**Background:**

We need to better understand the frequency and predictors of opioid use disorder (OUD) after first opioid prescription (OP).

**Methods:**

We followed 1 516 392 individuals from the Swedish population born 1980–2000, from 1 July 2007, until 31 Dec 2017. We examined putative risk predictors with univariable and multivariable Cox Models and the potential causal effects of predictors by propensity score and co-sibling analyses.

**Result:**

Of the individuals in our cohort, 24.8% (375 404) received a first OP, of whom 3034 (0.90%) developed a subsequent first OUD. The hazard ratio (HR) (± 95% CIs) for OUD after OP equaled 7.10 (6.75–7.46), with a mean time to onset of 3.41 (2.39) years. The strongest putative risk factors for development of OUD after OP were prior psychiatric and substance use disorders, criminal behavior, parental divorce/death, poor school performance, current community deprivation, divorce, and male sex. Few predictors differed across sexes. OP renewal was associated with a HR of 3.66 (3.41–3.93) for OUD. Co-sibling and propensity score analyses suggested that at least a moderate proportion of the risk factor-OUD association was likely causal. A risk score to predict OUD after OP had an AUC of 0.85, where nearly 60% of cases scoring in the top decile.

**Conclusions:**

In a general population sample, an OP represents a substantial risk factor for subsequent OUD. Many of the risk factors for OUD after OP can be readily assessed at the time of potential OP, permitting clinicians to evaluate the risk of iatrogenic OUD.

Opioid use disorder (OUD) is a public health problem of major proportions (Beauchamp, Winstanley, Ryan, & Lyons, 2014). Because a significant proportion of OUD develops after receipt of an opioid prescription (OP) (Edlund et al., 2014; Fishbain, Cole, Lewis, Rosomoff, & Rosomoff, 2008; Kolodny et al., 2015; Minozzi, Amato, & Davoli, 2013; Voon, Karamouzian, & Kerr, 2017), the indications for OP for treatment of pain has come under increased scrutiny with a need to balance the importance of pain control with the long-term risk of iatrogenic OUD (Sehgal, Manchikanti, & Smith, 2012). This public health importance of these issues has grown along with the large rise in rates of opioid use and opioid-related deaths in recent decades in most countries of Europe and North America including Sweden (Andersson, Håkansson, Krantz, & Johnson, 2020; Fugelstad, Thiblin, Johansson, Ågren, & Sidorchuk, 2019; Hastie, Gilson, Maurer, & Cleary, 2014).

In response to this urgent question, empirical studies have sought to predict OUD risk after OP (Boscarino et al., 2010; Cochran et al., 2014; Klimas et al., 2019; Reps, Cepeda, & Ryan, 2020), most of which have had modest sample sizes, samples of convenience, limited predictors and/or short follow-up periods (Beauchamp et al., 2014) (Minozzi et al., 2013). In this article, we attempt to address many of the methodological concerns of these earlier studies, examining five specific questions:

1. How much does receipt of an OP increase risk for subsequent OUD?
2. Explore, in univariable and multivariable analyses, a wide range of risk factors assessed at the time of OP that might predict development of OUD.
3. Determine whether duration of OP alters risk for subsequent OUD.
4. Perform propensity-score matching and co-sibling analyses to gain insight into the causal nature of the association between our predictors and OUD risk.
5. From multivariable analyses, develop, in a training split-half our sample, an aggregate risk score for OUD onset after OP and then, in the test split-half, evaluate its performance.

## Methods

We linked nationwide Swedish registers via the unique 10-digit identification number assigned at birth or immigration to all Swedish residents. The identification number was replaced by a serial number to ensure anonymity. We secured ethical approval for this study from the Regional Ethical Review Board of Lund University (No. 2008/409, 2012/795, and 2016/679).

The following sources were used to create our dataset: Total Population Register, containing information about year of birth, and sex; Multi-Generation Register, linking individuals born after 1932 to their parents; the Longitudinal Integration Database for Health Insurance and Labor Market Studies (LISA) containing information about education and income from 1990 to 2018; the Hospital Discharge Register, containing hospitalizations for Swedish inhabitants from 1964 to 2018; the Day Surgery Register, containing diagnosis from 1997 to 2000; the Prescribed Drug Register, containing all prescriptions in Sweden picked up by patients from July 2005 to 2018; Outpatient Care Register, containing information from all outpatient clinics from 2001 to 2018; Crime Register that included national complete data on all convictions in lower court from 1973–2018; Swedish Suspicion Register that included national data on individuals strongly suspected of crime from 1998–2018; and the Mortality Register with dates and causes of death from 1952 until 2018. In addition, we had medical diagnosis from Primary Health Care clinics from counties in Sweden which counties included 87% of the Swedish population [for details see (Sundquist et al., 2017)]. We included everyone born between 1980 and 2000 without a registration of OUD prior to July 2007.

### Measures

The outcome measure was OUD which we assessed using a medical registration of the ICD-10 code of F11 from any of the above registers, or from the Prescribed Drug Register using the ATC code representing prescribed drugs for opioid dependence; N07BC. In addition, we used criminal convictions from 1996 to 2006 and in 2009 when the type of drug used was registered. OPs were identified from the Prescribed Drug Register using ATC code for opioids; N02A.The outcome measure was OUD which we assessed using a medical registration of the ICD-10 code of F11 from any of the above registers, or prescribed drugs for opioid dependence; N07BC. In addition, we used criminal convictions from 1996 to 2006 and in 2009 when the type of drug used was registered. OPs were identified from the prescribed drug register using ATC code N02A. As we want to assess the first OP, we censored the first two years from the prescribed drug register to reduce the probability that we would count a repeat OP as a first OP. Thus, we only counted prescriptions after July 2007 when no prior opioids are prescribed. Using the Swedish Register Data, we assessed a number of possible predictors which are all outlined in online Supplementary Appendix Table 1.

### Statistical methods

We began by assessing the overall risk of OUD as a consequence of OP utilizing a Cox proportional Hazard model with first OP as a time dependent covariate. We follow individuals from July 2007 until first OUD diagnosis, death, emigration, or end of follow-up, whichever comes first. We estimated both the raw association and adjusted for fixed covariates including sex, parental education, parental divorce, educational achievement, and Familial Genetic Risk Score for drug use disorder (DUD) (FGRS_DUD_).

In the second set of analyses, we focused on individuals who have received an OP. Using Cox proportional hazard models we follow individuals from time of first OP until first OUD diagnosis, death, emigration, or end of follow-up.

We first estimate the overall raw associations for each of the predictors in the whole study sample, adjusting for sex. Next, we investigated whether the association differed between males and females by allowing for an interaction term between each predictor and sex. The results from this model were presented as HRs for males and females separately and in addition, the ratios between these ratios, representing the interactions, are presented. Finally, we included all predictors in a full, multivariable model. We investigated the proportionality assumption for the categorical variables visually by plotting the Kaplan–Maier curves. For the continuous variables we tested whether the HR was stable during follow-up time.

In a third set of analyses, we addressed the question of causality using two different approaches. First, we use propensity score matching (Austin, 2011) to construct groups that are as equal as possible with respect to the probability of having the exposure (an OP) and then we compare the risk of OUD for the group exposed compared to the ones unexposed, for each one of the predictors. We used the matchIt package in R and the nearest neighbor method to match individuals. Next, we use a co-sibling design that account for genetic and environmental factors shared within sibling pairs (Ohlsson & Kendler, 2020). Siblings discordant on the exposure and outcome, or the time of the outcome contribute to the analysis. To quantify the amount of the raw associations that can be explained be confounding factors, we compare the raw HRs with the HRs obtained from propensity score matching and co-sibling analysis. We compare the linear form of the parameters from the Cox regressions and present, as a percentage, the amount of the association that is explained by cofounding. For further background on these analyses, see online Supplementary Appendix Table 2.

In the final set of analysis to evaluate the predictive effect of our model we split the analytic sample into two random halves and run a logistic model, including all predictors, on the first or training half. Than we evaluate our model using the second, testing half of the sample by estimating the predictive probabilities of OUD given the parameter estimated from the training half and compare with the observed values. The results are presented as a ROC curve with the corresponding AUC value.

## Results

### Descriptive results

We began with a population sample of 1 516 392 individuals (51.7% males and 48.3% female) born from 1980–2000, followed from 1 July 2007, until 31 Dec 2017. During this follow-up period, 375 404 of them (24.8%) received a first OP, of whom 335 833 had complete data for analysis. After their first OP, 3034 of these individuals (0.90%) were registered for the first time for an OUD. The mean time (s.d.) from OP to OUD diagnosis in this sample was 3.41 (2.39) years and was modestly shorter in males [3.34 (2.35)] than in females [3.53 (2.46)] [*p* = 0.04]. The risk for OUD over the first 1, 2 and 5 years after first OP equaled 0.17, 0.32 and 0.68%, respectively.

### Impact of OP on OUD risk

The raw HR (± 95% CIs) for OUD conditional upon receipt of an OP was estimated (± 95% CIs) at 7.10 (6.75–7.46). When including key fixed covariates including sex, parental education, educational achievement and FGRS_DUD_, this HR decreased to 5.81 (5.49–6.15).

### Prediction of OUD – Univariable models

The frequency of our putative predictors of OUD and their inter-correlations are seen in online Supplementary Appendix Tables 3 and 4, respectively. Although the mean (s.d.) inter-correlation between them was modest [+0.16 (0.15)], some inter-correlations – for example between prior DUD, alcohol use disorder (AUD) and criminal behavior (CB) –were substantial. Table 1 provides the univariable analysis of our predictors in our entire sample in the left-hand column. Our putative predictors were divided into eight categories in rough chronological order of their occurrence: (i) demographic features, (ii) parental characteristics, (iii) genetic risk, (iv) educational performance, (v) prior psychiatric, and substance use disorders and CB, (vi) prior injuries and pain diagnoses, (vii) current marital status and (viii) community characteristics.

**Table 1.** Univariable, sex-specific and multivariable analyses of putative predictors of opiate use disorder after opiate prescription

		Univariable				
Categories of putative predictors	Putative predictor	entire sample	Males (HR, 95% CI)	Females (HR, 95% CI)	Male v. Female	Multivariable entire sample
Demographic features	Male v. Female	1.88 (1.74–2.02)			–	1.54 (1.41–1.68)
	Birth year	1.02 (1.02–1.03)	1.02 (1.01–1.03)	1.02 (1.01–1.04)	1.00 (0.98–1.02)	0.97 (0.94–0.98)
	Age at prescription	0.97 (0.97–0.98)	0.98 (0.97–0.99)	0.97 (0.96–0.98)	1.01 (0.99–1.03)	0.92 (0.90–0.94)
	Not Swedish born	0.94 (0.80–1.10)	1.08 (0.91–1.30)	0.65 (0.47–0.90)	1.67 (1.16–1.42)	0.64 (0.53–0.77)
Parental characteristics	Mid v. high parental education	1.64 (1.52–1.78)	1.64 (1.48–1.81)	1.64 (1.45–1.87)	1.00 (0.85–1.18)	1.09 (1.00–1.19)
	Low v. high parental education	2.01 (1.76–2.29)	2.12 (1.80–2.49)	1.82 (1.46–2.27)	1.16 (0.88–1.53)	1.01 (0.87–1.18)
	Parental divorce/death	2.43 (2.26–2.61)	2.44 (2.23–2.69)	2.41 (2.13–2.71)	1.02 (0.87–1.18)	1.15 (1.06–1.25)
Genetic risk	DUD FGRS (per s.d.)	1.31 (1.29–1.33)	1.31 (1.28–1.33)	1.33 (1.30–1.36)	0.98 (0.96–1.01)	1.11 (1.09–1.14)
Educational performance	Low grade (per s.d. unit)	1.92 (1.86–1.98)	1.92 (1.85–2.00)	1.91 (1.82–2.01)	1.01 (0.94–1.07)	1.29 (1.25–1.34)
Prior psychiatric and substance use disorders and CB	Prior AUD	7.79 (7.12–8.53)	7.53 (6.74–8.40)	8.39 (7.16–9.83)	0.90 (0.74–1.09)	1.68 (1.50–1.88)
	Prior CB	5.86 (5.45–6.31)	6.25 (5.70–6.84)	5.22 (4.61–5.91)	1.20 (1.03–1.40)	1.86 (1.69–2.05)
	Other prior DUD	12.59 (11.69–13.55)	11.75 (10.73–12.88)	14.25 (12.62–16.10)	0.82 (0.71–0.96)	4.70 (4.26–5.19)
	Prior DAD	5.17 (4.80 5.56)	5.54 (5.04–6.09)	4.65 (4.14–5.22)	1.19 (1.03–1.38)	2.74 (2.50–2.99)
	Prior bipolar or non-affective Psychotic disorders	5.69 (4.87–6.64)	6.89 (5.65–8.40)	4.48 (3.52–5.72)	1.54 (1.12–2.10)	1.38 (1.16–1.64)
Prior injuries and pain diagnoses	Prior pain diagnosis	1.37 (1.27–1.49)	1.35 (1.21–1.50)	1.41 (1.25–1.60)	0.95 (0.81–1.12)	1.11 (1.02–1.22)
	Prior injuries	1.16 (1.07–1.25)	1.10 (0.99–1.22)	1.23 (1.09–1.38)	0.89 (0.76–1.05)	0.83 (0.76–0.91)
Current marital status	Unmarried v. married	1.32 (1.13–1.53)	1.40 (1.12–1.75)	1.25 (1.12–1.75)	1.12 (0.83–1.52)	0.90 (0.75–1.06)
	Divorced/widowed v. married	1.98 (1.43–2.74)	2.02 (1.21–3.37)	2.02 (1.21–3.37)	1.05 (0.54–2.03)	1.00 (0.70–1.41)
Current community characteristics	Mid v. high community deprivation	1.58 (1.42–1.76)	1.57 (1.37–1.81)	1.59 (1.33–1.90)	0.99 (0.79–1.24)	1.16 (1.03–1.30)
	Low v. high community deprivation	2.57 (2.29–2.89)	2.58 (2.23–2.99)	2.57 (2.13–3.10)	1.00 (0.79–1.27)	1.24 (1.08–1.42)
	With DUD in neighborhood (by %)	1.24 (1.22–1.26)	1.23 (1.21–1.26)	1.26 (1.21–1.31)	0.98 (0.94–1.03)	1.03 (0.98–1.08)

Only one putative predictor – immigration status – was not significantly associated with OUD risk. The strongest risk factors were all in the ‘prior disorder’ category with HRs ranging from 5.17 (4.80 5.56) for major depression or anxiety disorders (DAD) to 12.59 (11.69–13.55) for prior DUD. Three predictors produced HRs in the range of 2–3: low v. high community deprivation and parental education and parental death/divorce. HRs between 1.5 and 2 were seen with male sex and poor grades in high school. The genetic risk had a relatively modest impact with a HR of 1.31 per s.d..

Table 1 also presents results for males and females separately and then, in the right-hand column, the difference between the two HRs. Only 4 predictors were significantly different, and all were more strongly associated in males: immigrant status, prior CB, DAD and bipolar or non-affective psychotic disorders.

While our registry data on the nature of the OP is limited and did not include the specific form of opioid and was often missing information about dosage, we could determine its renewal status in our entire sample. In a univariable analysis, renewal of the OP within six months was a robustly associated with future OUD with a HR of 3.66 (3.41–3.93). This HR, whose impact is over and above the 7-fold increased risk for OUD from the first OP, did not differ significantly in males and females, with a M/F ratio of 0.96 (0.83–1.11).

### Prediction of OUD – Multivariable models

The final column in Table 1 presents the multivariable results. As would be expected given the positive correlations between most of our predictors, all the HRs declined with four losing significance (low *v.* high parental education, unmarried *v.* married, divorced *v.* married and % of DUD in neighborhood). The five strongest multivariable predictors were, in order: prior DUD, prior DAD, prior CB, prior AUD and male sex.

### Causal effects

While casual effects of putative predictors cannot be evaluated definitively in observational data, we apply two distinct methods to provide insight into the degree to which our observed associations arise from causal effects *v.* confounders. Propensity Score Matching can be applied to all of our putative risk factors. Our co-sibling method, by contrast, cannot be utilized for risk factors for which full siblings are obligatorily concordant such as parental education or history of divorce. Furthermore, our co-sibling analyses can only be performed on sibling pairs concordant for OP exposure but discordant for the particular risk factor being evaluated. Even within our large sample size, such pairs were sometimes rare, which resulted in parameters estimated with poor precision.

Table 2 presents the results of these causal inference methods where we show the raw univariable findings which we then correct for sex. Next, we present the results from propensity score matching, from which we estimate the proportion of the association that is likely causal. Across all our predictors the propensity score matching, after eliminating the potentially protective effect of being non-Swedish born, the mean (s.d.) of the proportion of the association that is causal equaled 30.5% (25.6). Then we present the parallel results for the co-sibling analyses which estimates a considerably higher proportion of causal effects for our predictors: 62.9% (28.0). Because discordant co-siblings do not control completely for genetic confounding (sharing 50% of their genes identical by descent), the causal estimates from this method are likely biased upward.

**Table 2.** Analysis of potential causal effect of putative predictors by propensity score and co-sibling analyses

	Crude	Adjusted for sex	Matched by propensity score	Estimated percentage causal by propensity score	Co–sibling analysis^a^	Estimated percentage causal by co-sibling analysis
Parental divorce/death	2.20 (2.04. 2.38)	2.43 (2.26–2.61)	1.09 (1.01–1.17)	10		
Mid v. High community deprivation	1.51 (1.34–1.69)	1.58 (1.42–1.76)	1.35 (1.26–1.46)	66		
Low v. high community deprivation	2.37 (2.10–2.68)	2.57 (2.29–2.89)	1.28 (1.15–1.41)	26		
Unmarried v. married	1.49 (1.26–1.75)	1.32 (1.13–1.53)	1.29 (1.21–1.37)	93	1.55 (0.96–2.49)	100
Divorced/Widowed v. married	2.09 (1.48–2.95)	1.98 (1.43–2.74)	1.31 (0.82–2.08)	40	0.68 (0.24–1.94)	0
Low v. high parental education	1.87 (1.62–2.16)	2.01 (1.76–2.29)	0.96 (0.80–1.15)	0		
Mid v. high parental education	1.57 (1.45–1.71)	1.64 (1.52–1.78)	1.06 (0.98–1.13)	11		
Not Swedish born	0.92 (0.87.10)	0.94 (0.80–1.10)	0.60 (0.48–0.74)	–		--
Prior AUD	8.15 (7.38 9.00)	7.79 (7.12–8.53)	1.42 (1.24–1.65)	17	3.91 (2.47–6.20)	66
Other prior DUD	13.10 (12.13–14.14)	12.59 (11.69–13.55)	4.44 (3.91–5.04)	59	6.17 (4.25–8.95)	72
Prior CB	6.03 (5.58–6.51)	5.86 (5.45–6.31)	1.78 (1.61–1.96)	33	2.99 (2.20–4.06)	62
Prior DAD	4.30 (3.98–4.65)	5.17 (4.80 5.56)	1.99 (1.80–2.20)	32	2.73 (2.02–3.69)	61
Prior bipolar or non-affective Psychotic disorders	5.33 (4.50–6.31)	5.69 (4.87–6.64)	1.36 (1.09–1.74)	18	2.59 (1.28–5.25)	55
Pain diagnosis before	1.28 (1.17–1.40)	1.37 (1.27–1.49)	1.07 (0.96–1.19)	22	1.17 (0.88–1.55)	50
Injury diagnosis before	1.31 (1.22–1.42)	1.16 (1.07–1.25)	0.80 (0.76–0.85)	0	1.18 (0.90, 1.54)	100

a Results missing from co-sibling analyses for predictors that do not differ among siblings.

Both methods suggest that single marital status and prior DUD have strong causal effects on risk for OUD after OP with moderate to substantial causal effects seen for prior CB and DAD and at least modest causal impact for prior AUD, bipolar or non-affective psychotic disorders and pain diagnosis. We also examined the potential causal effect of renewal of the OP within 6 months which had a sex-adjusted HR of 3.66 (3.41, 2.93). The HR estimated from propensity score and co-sib analyses were 2.55 (2.32–2.81) and 1.56 (1.30–1.88), respectively, suggesting that 72% and 34% of its impact on OUD risk was likely causal.

### Development of predictive model

**Fig. 1.**
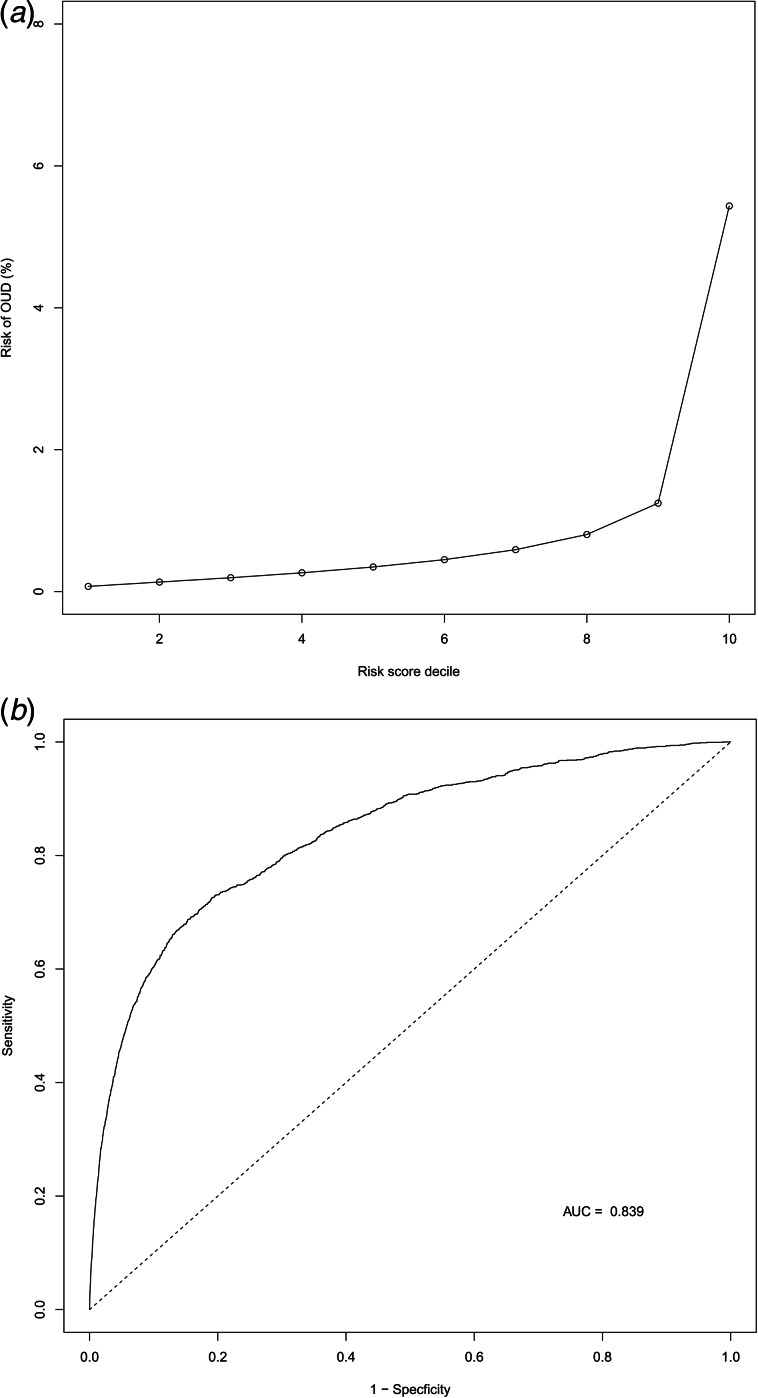
(a) The performance of our risk prediction model – The *Y* axis reflects the observed risk for OUD while the *X*-axis represents the decile of the risk score generated from our logistic regression model on the training split half of our sample and then applied to the testing split-half sample. (b) An AUC curve demonstrating the performance of our risk prediction model as applied to our testing split-half sample. The *Y*-axis represents the specificity of the model in predicting OUD while the *X*-axis represents 1- specificity.

We utilized 21 predictors which we examined in a multivariable logistic regression model predicting OUD in a random half of our sample to estimate beta parameters weights. We then applied those beta- parameters to the second half of our sample with the results displayed in Table 3. The pattern of findings results was, as expected, similar to that seen in the multivariable HR analyses in Table 1 with the strongest associations seen for prior DUD, DAD, CB, AUD and sex. As seen in Fig. 1a, 60% of all cases of OUD were found in the top decile of the risk score which has an OR per decile of 1.79 (1.73–1.84). For the AUC curve, equal to 0.84, see Fig. 1b.

**Table 3.** Prediction of OUD from a multivariable logistic regression confirmed on a second split-half of the sample

Predictor	OR (95% CI)
Male v. Female	1.44 (1.30–1.58)
Birth year	0.83 (0.82–0.84)
Age at prescription (by year)	0.77 (0.76–0.78)
Mid v. high parental education	1.12 (1.02–1.24)
Low v. high parental education	0.99 (0.82–1.17)
DUD grs (per s.d.)	1.06 (1.05–1.08)
Parental divorce/death	1.19 (1.09–1.31)
Low Grade (per s.d. unit)	1.35 (1.30–1.41)
Mid v. high community deprivation	1.05 (0.92–1.20)
Low v. high community deprivation	1.05 (0.90–1.23)
With DUD in neighborhood (by %)	1.01 (0.95–1.07)
Unmarried v. married	1.02 (0.83–1.26)
Divorced/widowed v. married	1.14 (0.74–1.70)
Not Swedish born	0.68 (0.55–0.84)
Prior AUD	1.64 (1.43–1.89)
Prior CB	1.99 (1.78–2.22)
Other prior DUD	4.73 (4.23–5.30)
Prior DAD	2.70 (2.43–3.00)
Prior bipolar or non-affective psychotic disorders	1.41 (1.12–1.75)
Prior pain diagnosis	1.12 (1.01–1.25)
Prior injuries	0.81 (0.73–0.89)

## Discussion

We addressed five questions in this paper all related to the delineation of risk predictors of the onset of OUD after a first OP. We review these results in turn.

*First*, we found that the increased risk for OUD association with an OP in the Swedish population was large, with a raw HR of 7.10 which was reduced to 5.86 with standard covariates. This is a much larger increased risk than recently seen in one-year follow-up of nearly 50 000 patients who received an OP as part of an ER visit (HR, 1.1; 95% CI 0.9–1.4) (Punches, Ancona, Freiermuth, Brown, & Lyons, 2021), and more modest than the nearly 15-fold increased risk (5.8 *v.* 0.4%) risk of an ‘opioid-abuse related diagnosis’ in a one year follow-up of a sample of over 750 000 adolescents and young adults from the Optum Research Database receiving a first time OP during a dental visit (Schroeder, Dehghan, Newman, Bentley, & Park, 2019). The magnitude of the OP-OUD association found in the Swedish general population highlights the public health importance of the risk for iatrogenic OUD. It also suggests that the risk for OUD after OP is not restricted to individuals whose OP is for the treatment of chronic pain.

We found a relatively low absolute risk for OUD after OP of 0.90%, considerably below the 4.7% obtained from a recent large-scale meta-analysis by Higgins et al. (Higgins, Smith, & Matthews, 2018). However, the rates varied widely across the 12 studies they reviewed, from 0.2% to 34.2% and most of the follow-up periods short, under one-year in duration (Higgins et al., 2018). Only one study they reviewed was a one-year prospective cohort design which found rates of 0.5% progression to OUD (Cepeda, Fife, Ma, & Ryan, 2013), about three-times higher than we observed over our first year of follow-up. Many of the studies reviewed by Higgins et al. (Higgins et al. 2018) focused on patients with chronic pain and this likely represents a group at considerably higher risk for OUD than our general population sample. As expected, our rates are considerably lower than those reported by Parker and Anthony for adolescents developing OUD within 12 months of starting to use prescription opioids extra-medically which ranged across ages and cohorts from 1.8% to 9.8% (Parker & Anthony, 2015).

*Second*, our univariable analyses revealed a number of risk factors for the development of OUD after OP from a range of different domains. Consistent with prior evidence, we found, in our univariable analyses, that the strongest predictors of development of OUD after OP was prior psychopathology (Boscarino et al., 2010; Burcher, Suprun, & Smith, 2018; Cochran et al., 2014; Hassan, Le Foll, Imtiaz, & Rehm, 2017; Rice et al., 2012) and substance use disorders (Burcher et al., 2018; Rice et al., 2012). Many of the other predictors of OUD development after OP that we observed in the Swedish population were supported by previous studies including being single (Burcher et al., 2018), low SES (Burcher et al., 2018), high familial risk for substance abuse (Burcher et al., 2018; Rice et al., 2012), being male (Burcher et al., 2018; Cochran et al., 2014), a young age at first OP (Burcher et al., 2018; Cochran et al., 2014), and having a pain disorder (Blanco et al., 2016; Cochran et al., 2014). When our analyses were repeated with a multivariable design, most HRs declined substantially, although the strongest predictors remained prior psychopathology and substance use disorders. These results highlight the multifactorial nature of the development of OUD after OP, consistent with our prior studies of all forms of DUD in Sweden (Kendler, Ohlsson, Edwards, Sundquist, and Sundquist, 2017).

We can particularly compare our results with those of a one year follow-up study of OUD after first OP in four large US data bases (Reps et al., 2020). Rates of OUD onset varied from 0.04–0.26% across cohorts, compatible with the one-year rate of 0.17% found in our sample. Furthermore, they generated an 8-item OUD predictor, six of which we replicated in our sample: young age, prior DUD, prior depression, prior anxiety disorder, and two medical conditions associated with chronic pain (Reps et al., 2020).

*Third,* in accord with prior studies, we found that longer opioid therapy was correlated with a substantially higher subsequent OUD risk (Cochran et al., 2014; Higgins et al., 2018).

*Fourth*, we utilized two different designs – co-sibling and propensity-score matching – to gain insight into the nature of the associations between our predictors of OUD risk after OP. These designs are quite different in their assumptions and potential limitations so that the joint use can be best seen as an example of triangulation (Munafo & Davey-Smith, 2018).These analyses are of importance for planning interventions based on putative risk factors as the reduction in caseness that might arise from such interventions would likely be closely related to the proportion of causal association between the risk factor and disease. We found reasonable but not complete agreement across our two methods with associations declining substantially across virtually all our predictors. In aggregate, across our entire range of predictors, as well as the strongest category of prior psychiatric and substance use disorders, approximately one third of the association appeared to be causal in its effects. We are not aware of prior studies attempting to predict rates of OUD after OP which have performed similar analyses.

*Fifth*, using a split-half method, we generated an aggregate risk score from our 21 predictor variables all of which could, theoretically, be assessed at the time at which a clinician is considering prescribing an opioid. The risk score had good sensitivity. Nearly 60% of all cases had scores in the highest decile and the area under the AUC curve equaled 0.85. However, the score had low specificity. In the highest risk decile, only 7% developed OUD. Many other assessment tools have been developed to predict risk for OUD after OP (Klimas et al., 2019). Only further empirical studies will enable a rigorous comparison of the utility and predictive ability of our measure.

How should the performance of our risk score be interpreted in the context of the results of our causal analyses? To our knowledge, such analyses have not been previously conducted on prior risk measures for OUD post OP (Klimas et al., 2019). Given the level of confounding that likely exists between our putative predictors and OUD, the expected benefits in reductions in OUD for interventions based on these risk factors would be less than expected from the raw HRs. That is, the risk score predictions will accurately predict caseness for OUD in a sample exposed to OP, but not all of that effect will be due to the OP. That is because a proportion of the subjects would have developed OUD in any event, given our evidence for confounding variables that predict both receipt of an OP and risk for OUD.

We found that 24.8% of our population based cohort had received and picked-up at least one OP during out 10 year follow-up period. Locating comparable data was difficult. We found one study based on the United States 2015 National Survey on Drug Use and Health, which estimated that 37.8% of their adult sample had used prescription opioids in the last year (Han et al., 2017). These results suggest that rates of OP in Sweden are likely substantially lower than those seen in recent years in the United States.

### Limitations

These results should be interpreted in the context of five potential methodological limitations.

First, our results are directly relevant only to the Swedish population although several factors suggest these findings likely generalize to other Western countries. We found substantial similarity in the risk factors for OUD that we identified and those reported in the prior literature. Furthermore, like the US, Sweden has seen increases in opioid consumption over recent decades (Hastie et al., 2014) as well as marked rises in opioid-related deaths (Andersson et al., 2020; Fugelstad et al., 2019). Like the US (Baldwin, Seth, & Noonan, 2021), there has been a shift in Sweden toward the use of short-acting opioids, especially oxycodone and fentanyl, with their high addictive potential and risk for overdose death (Baldwin et al., 2021; Heilig & Tägil, 2018) (Muller, Clausen, Sjøgren, Odsbu, & Skurtveit, 2019). Sweden, like the US, has substantial rates of non-medical use of prescription opioids (Han et al., 2017; Novak et al., 2016). Rates of OP in Sweden are in the range of those observed in US general population samples (Jeffery et al., 2018).

Second, while we detected subjects with DUD and OUD from medical, legal and pharmacy records which require neither respondent cooperation nor accurate recall, they can produce false negative and false positive diagnoses. While large interview studies of DUD prevalence are not available in Sweden, lifetime prevalence in near-by Norway was similar to the rates we detected in Sweden (Kringlen, Torgersen, & Cramer, 2001). It is not like that we substantially under-ascertain moderate to severe cases of DUD or OUD. The validity of our definition of DUD in Sweden is also supported by the high rates of concordance for registration observed across our different ascertainment methods (Kendler et al., 2015) and heritability estimates similar to those obtained from personal interview based studies (Kendler, Karkowski, Neale, & Prescott, 2000; Kendler, Maes, Sundquist, Ohlsson, & Sundquist, 2013; Tsuang et al., 1996).

Third, our estimates of the effect size of predictors of OUD and OP might be biased by baseline effects in which certain of these risk factors might have impacted on the clinician's decision to provide an OP in the first place.

Fourth, we do not have population level data on tobacco usage in Sweden, so are unable to model its impact on risk for OUD after OP.

Finally, we utilized a two-year period ‘buffer’ period to detect prior OPs which might be too short, resulting in some of our first OPs having previously received OPs. To evaluate this possible bias, we repeated our key analyses expanding the ‘buffer’ period to five years. As seen in online Supplementary Appendix Table 5, the pattern of predictors of OUD with 2 and 5 year buffer periods were very similar.

## Conclusion

In a Swedish national sample of individuals who received an OP for any indication, the risk for subsequent OUD was increased seven-fold. For those affected, the mean time from OP to first registration for OUD was 3.5 years. In univariate analyses, the development of OUD post OP was most strongly predicted by prior psychiatric and substance use disorders, a history of parental divorce, current community deprivation, divorce, poor prior school performance and male sex. A renewal of that first OP was associated with a 3.5-fold elevated risk for OUD. Co-sibling and propensity score analyses suggested a moderate to substantial proportion of the risk factor-OUD association was likely causal. Using split-half methods, we developed a predictive risk score with an AUC of 0.85, where nearly 60% of cases scored in the top decile. Our results document the public health importance of the substantial increase in OUD risk after OP and provide predictive models that permit clinicians to gauge, with at least moderate accuracy, individuals at particularly high risk for the development of OUD after OP.

## Supplementary material

For supplementary material accompanying this paper visit https://doi.org/10.1017/S003329172200349X. click here to view supplementary material

## References

[ref1] Andersson, L., Håkansson, A., Krantz, P., & Johnson, B. (2020). Investigating opioid-related fatalities in southern Sweden: Contact with care-providing authorities and comparison of substances. Harm reduction journal, 17(1), 1–11. 31918732 10.1186/s12954-019-0354-y PMC6953255

[ref2] Austin, P. C. (2011). An Introduction to propensity score methods for reducing the effects of confounding in observational studies. Multivariate Behavioral Research, 46(3), 399–424. doi: 10.1080/00273171.2011.568786 [doi] 21818162 PMC3144483

[ref3] Baldwin, G. T., Seth, P., & Noonan, R. K. (2021). Continued increases in overdose deaths related to synthetic opioids: Implications for clinical practice. Journal of the American Medical Association, 325, 1151–1152. 33571368 10.1001/jama.2021.1169 PMC9795485

[ref4] Beauchamp, G. A., Winstanley, E. L., Ryan, S. A., & Lyons, M. S. (2014). Moving beyond misuse and diversion: The urgent need to consider the role of iatrogenic addiction in the current opioid epidemic. American Journal of Public Health, 104(11), 2023–2029. 25211712 10.2105/AJPH.2014.302147 PMC4202970

[ref5] Blanco, C., Wall, M. M., Okuda, M., Wang, S., Iza, M., & Olfson, M. (2016). Pain as a predictor of opioid use disorder in a nationally representative sample. American Journal of Psychiatry, 173(12), 1189–1195. 27444794 10.1176/appi.ajp.2016.15091179

[ref6] Boscarino, J. A., Rukstalis, M., Hoffman, S. N., Han, J. J., Erlich, P. M., Gerhard, G. S., & Stewart, W. F. (2010). Risk factors for drug dependence among out-patients on opioid therapy in a large US health-care system. Addiction, 105(10), 1776–1782. 20712819 10.1111/j.1360-0443.2010.03052.x

[ref7] Burcher, K. M., Suprun, A., & Smith, A. (2018). Risk factors for opioid use disorders in adult postsurgical patients. Cureus, 10(5). 10.7759/cureus.2611 PMC6040780 30018867

[ref8] Cepeda, M. S., Fife, D., Ma, Q., & Ryan, P. B. (2013). Comparison of the risks of opioid abuse or dependence between tapentadol and oxycodone: Results from a cohort study. The Journal of Pain, 14(10), 1227–1241. 23850177 10.1016/j.jpain.2013.05.010

[ref9] Cochran, B. N., Flentje, A., Heck, N. C., Van Den Bos, J., Perlman, D., Torres, J., … Carter, J. (2014). Factors predicting development of opioid use disorders among individuals who receive an initial opioid prescription: Mathematical modeling using a database of commercially-insured individuals. Drug and Alcohol Dependence, 138, 202–208. 24679839 10.1016/j.drugalcdep.2014.02.701 PMC4046908

[ref10] Edlund, M. J., Martin, B. C., Russo, J. E., DeVries, A., Braden, J. B., & Sullivan, M. D. (2014). The role of opioid prescription in incident opioid abuse and dependence among individuals with chronic non-cancer pain: The role of opioid prescription. The Clinical Journal of Pain, 30(7), 557–564. 24281273 10.1097/AJP.0000000000000021 PMC4032801

[ref11] Fishbain, D. A., Cole, B., Lewis, J., Rosomoff, H. L., & Rosomoff, R. S. (2008). What percentage of chronic nonmalignant pain patients exposed to chronic opioid analgesic therapy develop abuse/addiction and/or aberrant drug-related behaviors? A structured evidence-based review. Pain Medicine, 9(4), 444–459. 18489635 10.1111/j.1526-4637.2007.00370.x

[ref12] Fugelstad, A., Thiblin, I., Johansson, L. A., Ågren, G., & Sidorchuk, A. (2019). Opioid-related deaths and previous care for drug use and pain relief in Sweden. Drug and Alcohol Dependence, 201, 253–259. 31260826 10.1016/j.drugalcdep.2019.04.022

[ref13] Han, B., Compton, W. M., Blanco, C., Crane, E., Lee, J., & Jones, C. M. (2017). Prescription opioid use, misuse, and use disorders in US adults: 2015 national survey on drug use and health. Annals of Internal Medicine, 167(5), 293–301. 28761945 10.7326/M17-0865

[ref14] Hassan, A. N., Le Foll, B., Imtiaz, S., & Rehm, J. (2017). The effect of post-traumatic stress disorder on the risk of developing prescription opioid use disorder: Results from the national epidemiologic survey on alcohol and related conditions III. Drug and Alcohol Dependence, 179, 260–266. 28818717 10.1016/j.drugalcdep.2017.07.012

[ref15] Hastie, B. A., Gilson, A. M., Maurer, M. A., & Cleary, J. F. (2014). An examination of global and regional opioid consumption trends 1980–2011. Journal of Pain & Palliative Care Pharmacotherapy, 28(3), 259–275. 25136898 10.3109/15360288.2014.941132

[ref16] Heilig, M., & Tägil, M. (2018). Do we have an opioid crisis in Scandinavia? Time to act? Acta Orthopaedica, 89(4), 368. 29714072 10.1080/17453674.2018.1465285 PMC6066774

[ref17] Higgins, C., Smith, B. H., & Matthews, K. (2018). Incidence of iatrogenic opioid dependence or abuse in patients with pain who were exposed to opioid analgesic therapy: A systematic review and meta-analysis. British Journal of Anaesthesia, 120(6), 1335–1344. 29793599 10.1016/j.bja.2018.03.009

[ref18] Jeffery, M. M., Hooten, W. M., Henk, H. J., Bellolio, M. F., Hess, E. P., Meara, E., … Shah, N. D. (2018). Trends in opioid use in commercially insured and medicare advantage populations in 2007–16: Retrospective cohort study. British Medical Journal, 362, 1–9. 10.1136/bmj.k2833 PMC6066997 30068513

[ref19] Kendler, K. S., Ji, J., Edwards, A. C., Ohlsson, H., Sundquist, J., & Sundquist, K. (2015). An extended Swedish national adoption study of alcohol use disorder. JAMA Psychiatry, 72(3), 211–218. doi: 2088151 [pii];10.1001/jamapsychiatry.2014.2138 [doi] 25565339 10.1001/jamapsychiatry.2014.2138 PMC4351126

[ref20] Kendler, K. S., Karkowski, L. M., Neale, M. C., & Prescott, C. A. (2000). Illicit psychoactive substance use, heavy use, abuse, and dependence in a US population-based sample of male twins. Archives of General Psychiatry, 57(3), 261–269. 10711912 10.1001/archpsyc.57.3.261

[ref21] Kendler, K. S., Maes, H. H., Sundquist, K., Ohlsson, H., & Sundquist, J. (2013). Genetic and family and community environmental effects on drug abuse in adolescence: A Swedish national twin and sibling study. American Journal of Psychiatry, 171(2), 209–217. 10.1176/appi.ajp.2013.12101300 PMC3927993 24077613

[ref22] Kendler, K. S., Ohlsson, H., Edwards, A. C., Sundquist, J., & Sundquist, K. (2017). A developmental etiological model for drug abuse in men. Drug and Alcohol Dependence, 179(July 29), 220–228. doi: 10.1016/j.drugalcdep.2017.06.036 28806639 PMC5623952

[ref23] Klimas, J., Gorfinkel, L., Fairbairn, N., Amato, L., Ahamad, K., Nolan, S., … Wood, E. (2019). Strategies to identify patient risks of prescription opioid addiction when initiating opioids for pain: A systematic review. JAMA Network Open, 2(5), e193365–e193365. 31050783 10.1001/jamanetworkopen.2019.3365 PMC6503484

[ref24] Kolodny, A., Courtwright, D. T., Hwang, C. S., Kreiner, P., Eadie, J. L., Clark, T. W., & Alexander, G. C. (2015). The prescription opioid and heroin crisis: A public health approach to an epidemic of addiction. Annual Review of Public Health, 36, 559–574. 10.1146/annurev-publhealth-031914-122957 25581144

[ref25] Kringlen, E., Torgersen, S., & Cramer, V. (2001). A Norwegian psychiatric epidemiological study. American Journal of Psychiatry, 158(7), 1091–1098. doi: 10.1176/appi.ajp.158.7.1091 11431231

[ref26] Minozzi, S., Amato, L., & Davoli, M. (2013). Development of dependence following treatment with opioid analgesics for pain relief: A systematic review. Addiction, 108(4), 688–698. 22775332 10.1111/j.1360-0443.2012.04005.x

[ref27] Muller, A. E., Clausen, T., Sjøgren, P., Odsbu, I., & Skurtveit, S. (2019). Prescribed opioid analgesic use developments in three Nordic countries, 2006–2017. Scandinavian Journal of Pain, 19(2), 345–353. 30677000 10.1515/sjpain-2018-0307

[ref28] Munafo, M. R., & Davey-Smith, G. (2018). Robust research needs many lines of evidence. Nature, 553(7689), 399–401. doi: d41586-018-01023-3 [pii];10.1038/d41586-018-01023-3 [doi] 10.1038/d41586-018-01023-3 32094809

[ref29] Novak, S. P., Håkansson, A., Martinez-Raga, J., Reimer, J., Krotki, K., & Varughese, S. (2016). Nonmedical use of prescription drugs in the European Union. BMC Psychiatry, 16(1), 1–12. 27488186 10.1186/s12888-016-0909-3 PMC4972971

[ref30] Ohlsson, H., & Kendler, K. S. (2020). Applying causal inference methods in psychiatric epidemiology: A review. JAMA Psychiatry, 77(6), 637–644. doi: 10.1001/jamapsychiatry.2019.3758 31825494 PMC7286775

[ref31] Parker, M. A., & Anthony, J. C. (2015). Epidemiological evidence on extra-medical use of prescription pain relievers: Transitions from newly incident use to dependence among 12–21 year olds in the United States using meta-analysis, 2002–13. PeerJ, 3, e1340. doi: 1310.7717/peerj.1340 26623183 10.7717/peerj.1340 PMC4662579

[ref32] Punches, B. E., Ancona, R. M., Freiermuth, C. E., Brown, J. L., & Lyons, M. S. (2021). Incidence of opioid use disorder in the year after discharge from an emergency department encounter. Journal of the American College of Emergency Physicians Open, 2(3), e12476. 34189517 10.1002/emp2.12476 PMC8219283

[ref33] Reps, J. M., Cepeda, M. S., & Ryan, P. B. (2020). Wisdom of the CROUD: Development and validation of a patient-level prediction model for opioid use disorder using population-level claims data. PLoS One, 15(2), e0228632. 32053653 10.1371/journal.pone.0228632 PMC7017997

[ref34] Rice, J. B., White, A. G., Birnbaum, H. G., Schiller, M., Brown, D. A., & Roland, C. L. (2012). A model to identify patients at risk for prescription opioid abuse, dependence, and misuse. Pain Medicine, 13(9), 1162–1173. 22845054 10.1111/j.1526-4637.2012.01450.x

[ref35] Schroeder, A. R., Dehghan, M., Newman, T. B., Bentley, J. P., & Park, K. (2019). Association of opioid prescriptions from dental clinicians for US adolescents and young adults with subsequent opioid use and abuse. JAMA Internal Medicine, 179(2), 145–152. 30508022 10.1001/jamainternmed.2018.5419 PMC6439650

[ref36] Sehgal, N., Manchikanti, L., & Smith, H. S. (2012). Prescription opioid abuse in chronic pain: A review of opioid abuse predictors and strategies to curb opioid abuse. Pain Physician, 15(3 Suppl), ES67–ES92. 22786463

[ref37] Sundquist, J., Ohlsson, H., Sundquist, K., & Kendler, K. S. (2017). Common adult psychiatric disorders in Swedish primary care (where most mental health patients are treated). BMC Psychiatry, 17, 1–9. 28666429 10.1186/s12888-017-1381-4 PMC5493066

[ref38] Tsuang, M. T., Lyons, M. J., Eisen, S. A., Goldberg, J., True, W., Lin, N., … Eaves, L. (1996). Genetic influences on DSM-III-R drug abuse and dependence: A study of 3372 twin pairs. American Journal of Medical Genetics, 67(5), 473–477. Retrieved from http://www.ncbi.nlm.nih.gov/pubmed/8886164. 8886164 10.1002/(SICI)1096-8628(19960920)67:5<473::AID-AJMG6>3.0.CO;2-L

[ref39] Voon, P., Karamouzian, M., & Kerr, T. (2017). Chronic pain and opioid misuse: A review of reviews. Substance Abuse Treatment, Prevention, and Policy, 12(1), 1–9. 28810899 10.1186/s13011-017-0120-7 PMC5558770

