# Supplementary material for: Risk factors for the development of opioid use disorder after first opioid prescription: a Swedish national study
Source: Psychol Med. 2022 Nov 23;53(13):6223–31. doi: 10.1017/S003329172200349X (PMC10520598; doi:10.1017/S003329172200349X)
Supplement: Supplementary file 1 [file S003329172200349Xsup001.docx]

Appendix for “Risk Factors for the Development of Opioid Use Disorder After Opioid Prescription: A Swedish National Study”

Table 1 - Data sources

The following sources were used to create our dataset: Total Population Register, containing information about year of birth, and sex; the Multi-Generation Register, linking individuals born after 1932 to their parents; the Longitudinal Integration Database for Health Insurance and Labor Market Studies (LISA) containing information about education and income from 1990 to 2018; the Hospital Discharge Register, containing hospitalizations for Swedish inhabitants from 1964 to 2018; the Day Surgery Register, containing diagnosis from 1997 to 2000; the Prescribed Drug Register, containing all prescriptions in Sweden picked up by patients from July 2005 to 2018; the Outpatient Care Register, containing information from all outpatient clinics from 2001 to 2018; the Crime Register that included national complete data on all convictions in lower court from 1973-2018; the Swedish Suspicion Register that included national data on individuals strongly suspected of crime from 1998-2018; and the Mortality Register with dates and causes of death from 1952 until 2018. In addition, we had medical diagnosis from Primary Health Care clinics from the following counties in Sweden; Blekinge (2009 - 2016), Dalarna (2005 - 2013), Värmland (2005 - 2015), Kalmar Län (2007 - 2016), Sörmland (1992 - 2017), Uppsala Län (2005 - 2015), Västernorrland (2008 - 2015) Norrbotten Län (2001 - 2014), Gävleborg (2010 - 2017), Gotland (2011 – 2018), Halland (2007 - 2014), Jönköpings Län (2008 - 2014), Kronoberg (2006 - 2016), Skåne (1989 - 2018), Västerbotten (1992- 2018), Östergötland (1990 - 2014), Stockholms Län (2003 - 2016), and Västra Götaland (2000 - 2013). The time-periods vary due to the regions’ different timing of digitalizing of the patient records.

Predictors

Parental education; highest level of both parent’s education and categorized into high (university level), mid (upper secondary school) and low (compulsory school only).

Parental separation; defined as living with both biological parents at age 16.

Genetic risk variable; FGRS_DUD_ further described in^1,2^. To calculate the FGRS-DUD we used all unique relatives with a registration for the disorder, and first non-parametrically estimated the distribution of age at first registration. The empirical distribution was used to obtain weights for relatives without a registration for the disorder, in order to account for the proportion of the time-at-risk period they had completed at the end of follow-up. For example, for relatives at age x at the end of follow-up, the weight corresponds to the proportion of relatives registered for the trait that had been registered at age x. For relatives born prior to 1958 we subtracted age at the end of follow-up with the following formula: 1958 - year of birth of relative. This modification was done in order to control for registration effects (i.e., most registers in Sweden start in 1973 suggesting that relatives from early birth cohorts do not have the possibility to be registered at younger ages). Note that all relatives with the disorder are weighted one. Second, the binary variable (DUD yes/no) was transformed into a z-score based on the threshold for DUD. The underlying liability of the individual is not assessable. Instead, we estimated the mean of the underlying liability to obtain sex and birth decade specific Z-scores for relatives with DUD registration and relatives without. We generated n random numbers from a N(0, 1) distribution and estimate the mean for relatives registered with the disorder (i.e., mean of the observations above the threshold) and for relatives without a registration (i.e., mean of all observation below the threshold). The thresholds are calculated for each decade of birth and sex. The third step was to correct for cohabitation effects. To estimate the cohabitation effect (i.e., “shared environment”), we created a dataset with all individuals in the Swedish population born in Sweden 1955-1990. We also included the number of years, during ages 0-15, that individuals resided in the same household as their biological father. We thereby were able to define two kinds of families: i) “not-lived-with” father families (offspring never resided for more than 1 year in the same household or in the same community as their biological father); ii) “lived-with” father (offspring resided a minimum of 13 year in the same household as their biological father. We performed a logistic regression model with the binary trait in offspring as outcome and the binary trait in father, type of father, and their interaction as predictors. We used the interaction term as the difference of effect between genes only and genes + environment. The same approach was performed for half-siblings where we compared those who were reared together versus reared apart. The following interaction terms were used in the calculations for DUD: Parent/Children = .92 and Siblings = .52. Fourth, we calculated the product for each relative using the four components: i) Z-score (reflecting sex and year of birth adjusted rates); ii) Weight (reflecting the proportion of risk period they had completed); iii) Cohabitation effects; iv) Proportion of shared genetic effects (0.03125 – 0.5) with the proband. Fifth, we averaged the product calculated in step 4 across all relatives to a proband. Sixth, we correct for the number of relatives. We multiplied the results from step 5 with a shrinkage factor. Shrinkage factor (SF): B/(B+A/C). It produces more shrinkage if B and C are small, and A is large. (A) the variance of the z-score of the disorder across all relatives, (B) the variance in the mean z-score across all probands, (C) the weighted number of relatives for each proband (sum of Column 3 across each proband). Seventh, we corrected for differences by year of birth and county differences. There are 21 counties in Sweden. For each proband we used the county they had resided in during the maximum number of years (measured from 1969 and onwards) We standardized the risk score by year of birth and county of the proband into a z-score with mean 0 and SD 1. This was then used as the FGRS_DUD_ in the analyses.

Educational performance was assessed at grade nine, the last year of compulsory school in Sweden (typically age 16) and standardized by year and gender.

Marital Status Marital status was assessed at the time of prescription and divided into the four categories; unmarried, married, divorced, and widowed.

Community Characteristics Neighborhood level socioeconomic status was also assessed at the time of prescription and based on Small Areas for Market Statistics (SAMS) obtained from Statistics Sweden. The average population is around 1000 inhabitants. We assessed the socioeconomic characteristics of each neighborhood using an aggregated measure, described in detail elsewhere^3^. The aggregated measure was based on four dimensions of deprivation in the working population aged 25–64; the proportion of people with low income, low education, unemployment, and receipt of social welfare and standardized to have mean 0 and standard deviation 1 each year. Based on the standardized score the socioeconomic level of each neighborhood was categorized as low (deprivation score above 1), mid (deprivation score between -1 and 1) or high (deprivation score below -1).

AUD status was identified from Swedish medical registries by the following ICD codes: ICD8: 571.0, 291, 303, 980; ICD9: V79B, 305A, 357F, 571A, 571B, 571C, 571D, 425F, 535D, 291, 303, 980; and ICD 10: E244, G312, G621, G721, I426, K292, K700, K701, K702, K703, K704, K709, K852, K860, O354, T510, T511, T512, T513, T518, T519, F101, F102, F103, F104, F105, F106, F107, F108, F109; and from the Prescribed Drug Register if prescribed disulfiram (Anatomical Therapeutic Chemical (ATC) Classification System N07BB01), acamprosate (N07BB03), or naltrexone (N07BB04). In addition, we identified AUD as convicted for, or suspected of at least two alcohol-related crimes according to law 1951:649, paragraph 4 and 4A and law 1994:1009, Chapter 20, paragraph 4 and 5 from the Swedish Crime Register, and code 3005 and 3201 in the Suspicion register.

Criminal behavior (CB) was defined from the Swedish crime register and included violent crime, white collar crime or property crime as defined elsewhere^4^. Drug abuse other than OUD was from medical registers, DUD was identified using codes from ICD-9: 292, 304, and 305C - 305I; ICD-10: F12-F16 and F18-F19; in the Suspicion Register by codes 3070, 5010, 5011, and 5012; and in the Crime Register by references to law 1968:64, paragraph 1, point 6 or and law 1951:649, paragraph 4, subsection 2 and paragraph 4A, subsection 2. Depression and Anxiety (DAD) was defined using ICD-8: 296.0, 296.2, 298.0, 300.4, 300.0, 300.2, ICD-9; 296B, 298A, 300A, 300C, 300E and ICD-10: F32, F33, F40, F41. Bipolar disorder and other non-affective psychosis (BPN) were defined using, ICD-8; 295, 297, ICD-9; 295, 297, 298E, 298W, 298X, 296A, 296C, 296D, 296E, 296W, 298B, and ICD-10; F20, F22, F23, F24, F25, F26, F27, F28, F29, F30, F31. From Medical Registers we defined pain using the ICD-10 codes: R52, M50, M51, M53, M54 and injuries using ICD-10: S00-S99 and T00-T14.

Depression and Anxiety was identified using the following ICD codes: ICD8: 296.0, 296.2, 298.0, 300.4, 300.0, 300.2, ICD9: 296B, 298A, 300A, 300C, 300E, and ICD10: F32, F33, F40, F41.

Bipolar disorder and other non-affective was identified using ICD8: 295, 297, ICD9: 295, 297, 298E, 298W, 298X, 296A, 296C, 296D, 296E, 296W, 298B, and ICD10: F20, F22, F23, F24, F25, F26, F27, F28, F29, F30, F31.

Pain related diagnosis was identified using the ICD-10 codes; R52, M50, M51, M53, M54.

Injuries using ICD10: S00-S99, and T00-T14.

References

1. Kendler KS, Ohlsson H, Sundquist J, Sundquist K. Family Genetic Risk Scores and the Genetic Architecture of Major Affective and Psychotic Disorders in a Swedish National Sample. *JAMA Psychiatry.* 2021;78:735-743.

2. Kendler KS, Ohlsson H, Sundquist J, Sundquist K. The patterns of family genetic risk scores for eleven major psychiatric and substance use disorders in a Swedish national sample. *Transl Psychiatry.* 2021;11(1):1-8.

3. Winkleby M, Sundquist K, Cubbin C. Inequities in CHD incidence and case fatality by neighborhood deprivation. *American Journal of Preventive Medicine.* 2007;32(2):97-106.

4. Kendler KS, S. LL, Morris NA, Sundquist J, Långström N, Sundquist K. A Swedish National Adoption Study of Criminality. *Psychol Med.* 2014;44(9):1913-1925.

Table 2 Frequency of Putative Predictors

|  | (col%) | Rates of OUD (row %) |
| --- | --- | --- |
| In total | 335,833 | 3,034 (0.90%) |
| Males | 156,644 (46.64%) | 1,881 (1.20%) |
| Females | 179,189 (53.36%) | 1,153 (0.64%) |
| No parental separation | 202,411 (60.27%) | 1,183 (0.58%) |
| Parental divorce/death | 133,422 (39.73%) | 1,851 (1.39%) |
| High NSES | 73,676 (21.94%) | 399 (0.54%) |
| Mid NSES | 188,333 (56.08%) | 1,609 (0.85%) |
| Low NSES | 73,824 (21.98%) | 1,026 (1.39%) |
| Unmarried | 296,883 (88.40%) | 2,810 (0.95%) |
| Married | 34,177 (10.18%) | 178 (0.52%) |
| Divorced/ Widowed | 4,773 (1.42%) | 46 (0.96%) |
| Low parental education | 22,465 (6.69%) | 300 (1.34%) |
| Mid parental education | 170,832 (50.87%) | 1,801 (1.05%) |
| High parental education | 142,536 (42.44%) | 933 (0.65%) |
| Swedish born | 316,868 (94.35%) | 2,868 (0.91%) |
| Not Swedish born | 18,965 (5.65%) | 166 (0.88%) |
| No AUD before | 325,411(96.90%) | 2,446 (0.75%) |
| AUD before | 10,422 (3.11%) | 588 (5.64%) |
| No other DUD before | 311,327 (92.70%) | 1,574 (0.51%) |
| Other DA before | 24,506 (7.30%) | 1,460 (5.95%) |
| No CB before | 293,408 (87.38%) | 1,609 (0.55%) |
| CB before | 42,425 (12.63%) | 1,425 (3.36%) |
| No DAD before | 279,269 (83.16%) | 1,761 (0.63%) |
| DAD before | 56,564 (16.84%) | 1,273 (2.25%) |
| No BPN before | 331,179 (98.61%) | 2,862 (0.86%) |
| BPN before | 4,654 (1.39%) | 172 (3.70%) |
| No pain diagnosis before | 254,360 (75.74%) | 2,221 (0.87%) |
| Pain diagnosis before | 81,473 (24.26%) | 813 (1.00%) |
| No injury diagnosis before | 113,286 (33.73%) | 923 (0.81%) |
| Injury diagnosis before | 222,547 (66.27%) | 2,111 (0.96%) |
| Low Grade, mean (SD) | 0.18 (1.01) | 0.98 (1.14) |
| DA grs (per 0.01 units) | -0.01 (1.00) | 0.68 (1.67) |
| Start age | 24.55 (4.75) | 23.09 (3.95) |
| DUD in neighborhood (%) | 0.66 (0.58) | 0.86 (1.2) |
| No renewal within 6 months | 250,037 (74.45%) | 1443 (0.58%) |
| Renewal within 6 months | 85,796 (25.55%) | 1,591 (1.85%) |

| Table 3  Inter-correlations between the Predictors of OUD after OP | | | | | | | | | | | | | |
| --- | --- | --- | --- | --- | --- | --- | --- | --- | --- | --- | --- | --- | --- |
|  | *Parental education* | *Parental separation* | *NSES* | *Foreign born* | *AUD before* | *DUD before* | *CB before* | *DAD before* | *BPN before* | *Pain diagnosis before* | *Injury before* | *Low SA* | *DUD grs* |
| *Parental education* | 1 (0) | 0.113 (0.003) | 0.225 (0.002) | 0.138 (0.004) | 0.108 (0.005) | 0.126 (0.004) | 0.186 (0.003) | 0.049 (0.003) | -0.016 (0.007) | 0.098 (0.003) | 0.005 (0.003) | 0.369 (0.002) | 0.157 (0.002) |
| *Parental separation* |  | 1 (0) | 0.124 (0.002) | -0.207 (0.004) | 0.222 (0.005) | 0.269 (0.004) | 0.244 (0.003) | 0.178 (0.003) | 0.130 (0.007) | 0.049 (0.003) | 0.021 (0.003) | 0.271 (0.002) | 0.234 (0.002) |
| *Low NSES* |  |  | 1 (0) | 0.322 (0.004) | 0.102 (0.005) | 0.171 (0.004) | 0.163 (0.003) | 0.098 (0.003) | 0.092 (0.006) | 0.072 (0.003) | 0.006 (0.003) | 0.220 (0.002) | 0.130 (0.002) |
| *Foreign born* |  |  |  | 1 (0) | -0.063 (0.009) | 0.134 (0.006) | 0.210 (0.005) | -0.023 (0.005) | -0.035 (0.012) | 0.111 (0.005) | -0.068 (0.004) | 0.143 (0.003) | 0.114 (0.002) |
| *AUD before* |  |  |  |  | 1 (0) | 0.603 (0.004) | 0.475 (0.005) | 0.424 (0.005) | 0.399 (0.009) | 0.063 (0.006) | 0.176 (0.006) | 0.270 (0.004) | 0.131 (0.003) |
| *DUD before* |  |  |  |  |  | 1 (0) | 0.643 (0.003) | 0.332 (0.004) | 0.394 (0.007) | 0.058 (0.004) | 0.195 (0.004) | 0.343 (0.003) | 0.204 (0.002) |
| *CB before* |  |  |  |  |  |  | 1 (0) | 0.190 (0.004) | 0.205 (0.008) | 0.067 (0.004) | 0.157 (0.004) | 0.374 (0.002) | 0.200 (0.002) |
| *DAD before* |  |  |  |  |  |  |  | 1 (0) | 0.627 (0.005) | 0.227 (0.003) | 0.005 (0.003) | 0.187 (0.002) | 0.097 (0.002) |
| *BPN before* |  |  |  |  |  |  |  |  | 1 (0) | 0.096 (0.008) | 0.034 (0.007) | 0.121 (0.005) | 0.079 (0.004) |
| *Pain diagnosis before* |  |  |  |  |  |  |  |  |  | 1 (0) | 0.069 (0.003) | 0.109 (0.002) | 0.043 (0.002) |
| *Injury before* |  |  |  |  |  |  |  |  |  |  | 1 (0) | 0.028 (0.002) | 0.005 (0.002) |
| *Low SA* |  |  |  |  |  |  |  |  |  |  |  | 1 (0) | 0.203 (0.198) |
| *DUD grs* |  |  |  |  |  |  |  |  |  |  |  |  | 1(0) |

| Table 4  A Comparison of Univariable Prediction of OUD and OP With two and Five Year Censoring Windows | | | |
| --- | --- | --- | --- |
| Categories of Putative Predictors | Putative Predictor | Entire Sample - Univariable | |
|  |  | Two year censoring | Five year censoring |
| Demographic Features | Male vs Female | 1.88 (1.74, 2.02) | 1.97 (1.78, 2.18) |
|  | Birth year | 1.02 (1.02, 1.03) | 1.04 (1.03, 1.05) |
|  | Age at prescription | 0.97 (0.97, 0.98) | 0.96 (0.95, 0.97) |
|  | Not Swedish born | 0.94 (0.80, 1.10) | 0.93 (0.75, 1.16) |
| Parental Characteristics | Mid vs high parental education | 1.64 (1.52, 1.78) | 1.52 (1.36, 1.69) |
|  | Low vs high parental education | 2.01 (1.76, 2.29) | 2.07 (1.74, 2.46) |
|  | Parental divorce/death | 2.43 (2.26, 2.61) | 2.39 (2.16, 2.64) |
| Genetic Risk | DUD grs (per SD) | 1.31 (1.29, 1.33) | 1.31 (1.28, 1.33) |
| Educational Performance | Low grade (per SD unit) | 1.92 (1.86, 1.98) | 1.95 (1.87, 2.04) |
| Prior Psychiatric and Substance Use Disorders and Criminal Behavior | Prior AUD | 7.79 (7.12, 8.53) | 7.93 (7.04, 8.93) |
|  | Prior CB | 5.86 (5.45, 6.31) | 6.03 (5.45, 6.66) |
|  | Other prior DUD | 12.59 (11.69, 13.55) | 13.69 (12.36, 15.15) |
|  | Prior DAD | 5.17 (4.80 5.56) | 5.50 (4.98, 6.07) |
|  | Prior BPN | 5.69 (4.87, 6.64) | 5.08 (4.15, 6.22) |
| Prior Injuries and Pain Diagnoses | Prior pain diagnosis | 1.37 (1.27, 1.49) | 1.41 (1.27, 1.56) |
|  | Prior injuries | 1.16 (1.07, 1.25) | 1.26 (1.12, 1.41) |
| Current Marital Status | Unmarried vs married | 1.32 (1.13, 1.53) | 1.63 (1.32, 2.00) |
|  | Divorced/widowed vs married | 1.98 (1.43, 2.74) | 2.53 (1.71, 3.76) |
| Current Community Characteristics | Mid vs high Community Deprivation | 1.58 (1.42, 1.76) | 1.60 (1.38, 1.86) |
|  | Low vs high Community Deprivation | 2.57 (2.29, 2.89) | 2.66 (2.27, 3.12) |
|  | With DUD in neighborhood (by %) | 1.24 (1.22, 1.26) | 1.24 (1.21, 1.27) |
